# CITED Proteins in Cardiac Development and Lifelong Heart Function

**DOI:** 10.3390/jpm15110542

**Published:** 2025-11-07

**Authors:** José Bragança, Rute Luísa Cabrita Pinto, Igor Ventura, Silvana Ferreira, António Marreiros

**Affiliations:** 1 Faculty of Medicine and Biomedical Sciences (FMCB), Campus Gambelas, University of Algarve, 8005-139 Faro, Portugal; ruteluisapinto@gmail.com (R.L.C.P.); a70941@ualg.pt (I.V.); a74779@ualg.pt (S.F.); a87786@ualg.pt (A.M.); 2 Algarve Biomedical Center-Research Institute (ABC-RI), Campus Gambelas, University of Algarve, 8005-139 Faro, Portugal; 3 Centre for Health Technology and Services Research, Health Research Network (CINTESIS@RISE), University of Algarve, 8005-139 Faro, Portugal

**Keywords:** CITED1, CITED2, CITED4, heart development, heart diseases, congenital heart disease

## Abstract

The CITED proteins function as transcriptional modulators that are essential for vertebrate development. These proteins interact with numerous partners, notably transcription factors and co-activators. The hallmark of the CITED family is their conserved carboxy-terminal domain, which interacts strongly with the CBP/p300 co-activators. The expression of CITED genes is detected early during embryogenesis within embryonic and foetal regions critical for cardiac morphogenesis, among other developmental processes. Notably, CITED2 loss of function is strongly associated with congenital heart malformations in mice and zebrafish embryos, as well as congenital heart disease (CHD) in humans, whereas other CITED family members are not critical for cardiogenesis. Emerging evidence implicates CITED2 and CITED4 in regulating heart physiological adaptations and protective responses to pathological stress. This review provides a detailed analysis of CITED proteins and their interactors, focusing on CITED-target genes relevant for cardiogenesis and heart disease. We also highlight recent findings indicating that CITED2 and CITED4 may be instrumental for the development of novel therapeutic strategies to mitigate CHD and preserve adult cardiac function.

## 1. Introduction

Congenital heart diseases (CHDs) are structural and functional anomalies resulting from improper foetal development present in nearly 1% of live births, with an estimated 2–5% risk of familial recurrence. As infant mortality rates decrease, more adults with complex CHDs are surviving and are at risk for severe cardiovascular complications [1]. Many children and adults with CHDs require surgeries and lifelong medical care, but inequalities in access to quality CHD care persist worldwide [2,3]. Mutations in genes encoding for transcriptional and epigenetic/chromatin remodelling factor, cell signalling, adhesion and structural sarcomere proteins, as well as chromosomal abnormalities can disrupt heart development, leading to CHDs [1]. Additionally, inadequate placental function, sporadic developmental errors, and/or maternal environmental stressors, such as diabetes, some medications and drugs abuse, infections, tobacco smoke, alcohol, and harmful chemicals, increase the risk of CHDs [1]. Despite scientific and clinical advancements, CHDs remain a significant global health and economic burden. Non-congenital heart conditions, collectively termed acquired cardiovascular diseases (ACDs), represent a spectrum of common heart disorders that develop after birth, which include coronary artery disease and rheumatic heart disease, among others. ACDs are the accountable cause for more than 30% of deaths worldwide annually. Ischemic (coronary) heart disease remains the dominant cause among cardiovascular deaths, and the burden is significant across all countries and economical levels, although over three quarters of these deaths are recorded in countries with emerging economies. The continuing evolution of ACD epidemiology highlights the need for prevention, screening, and intervention strategies adapted to regional risk profiles and health system capacities [4].

The CBP/p300-interacting transactivator with ED-rich tail (CITED) family includes transcriptional regulators present only in vertebrates that interact strongly with the acetyltransferases and transcriptional co-activators CBP and p300 [5,6,7,8]. During mouse embryogenesis, CITED proteins display diverse functions and are crucial for multiple developmental and morphological processes. Among the CITED members, Cited2 is currently the most extensively studied and has been demonstrated to be indispensable for mouse embryonic survival and growth during gestation, as well as for the correct development of the heart, placenta, and other organs [5,9,10,11,12,13,14,15,16,17]. In humans, abnormal regulation and mutations of *CITED2* have been correlated to CHDs [1]. On the other hand, despite *Cited1* being expressed in key cardiogenic structures and present in embryonic mouse hearts, its role in cardiac development is less critical compared to *Cited2*. *Cited4* is not essential for mouse embryo development, but in adult rodent hearts, its expression is linked to physiological cardiac hypertrophy induced by exercise training [18,19]. *Cited3* is the last member of the *CITED* genes, being expressed exclusively in birds and fish, where it also plays a role in their normal developmental processes [20,21].

Strong evidence suggests that the self-renewal and differentiation defects observed in *Cited2*-deficient mouse embryonic stem cells (ESCs), along with the lethality and multiple organ malformations in *Cited2*-null mouse embryos, can be rescued through supplementation or ectopic expression of human CITED2 protein or other Cited2-regulated proteins, including Wnt5a and Wnt11 [22,23,24,25]. Like in mice, *Cited2* depletion from the one-cell stage in zebrafish embryos increased early embryonic death and cardiovascular defects, which were substantially rescued following microinjection of recombinant human CITED2 or Wnt5a/Wnt11 proteins [24]. Therefore, the human CITED2 protein exhibits functional equivalence with the endogenous Cited2 proteins of mice and zebrafish, supporting the notion that CITED2 activity is evolutionarily conserved among vertebrates. In the adult heart, *Cited2* and *Cited*4 fulfil distinct yet critical functions for cardiac homeostasis, supporting exercise-induced adaptation and conferring protection against cardiovascular stress and pathology, while *Cited1* plays a minimal role in these mechanisms [6,18,26,27,28].

In this review, we discuss the roles of CITED family members in cardiogenesis and adult cardiac function, as well as their associations with heart disease. We report advances in understanding the molecular and cellular functions of *Cited* genes, which are essential for elucidating their contributions to normal heart development, homeostasis, and pathology. We also highlight recent research methods and techniques employed to extend the understanding of CITED functions, which could contribute to the development of novel therapeutic strategies to prevent or manage cardiac dysfunction.

## 2. Heart Development

**Figure 1:**
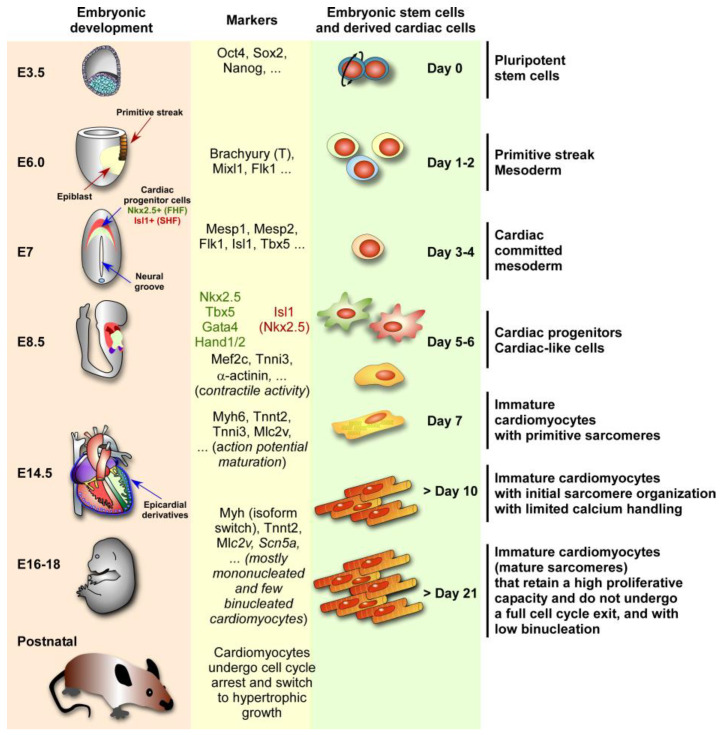
Mouse embryonic development compared to embryonic stem cell cardiac differentiation. Left panel: cardiac structures and timings in embryonic days (E) of mouse embryonic development until birth. Middle panel: some markers specific to each step of heart development and cardiac differentiation. Right panel: steps and timings of cardiac differentiation from mouse embryonic stem cells (pluripotent stem cells). Note that the cardiac differentiation from ESCs and heart embryonic development follow similar steps and share similar molecular markers. At E3.5, the blastocyst has an inner cell mass (blue cells) from which embryonic pluripotent stem cells are derived, which are characterized by the expression of Nanog, Oct4, and Sox2, among other genes. At E6.0, the formation of the primitive streak initiates, which initiates gastrulation and the generation of the mesoderm. At E7.0, the cardiac crescent is formed, composed of cardiac progenitor cells of the first heart field (FHF) marked by the expression of Nkx2.5 (green field) and the secondary heart field (SHF) marked by the expression of Isl1 (red field), after specification of some cells of the mesoderm into the cardiac mesoderm marked by Mesp1 expression. At 8.5, the linear heart tube is formed, and cardiac contractile activity is detected. By E14.5, the heart is formed by 4 chambers with cells of the FHF contributing to the left ventricle and atrium, and cells of the SHF contributing to the right ventricle and atrium. Epicardial cells will derive from the epicardium. After birth, many cardiomyocytes undergo one final nuclear mitosis without complete cell division, leading to multinucleation (cells with two or more nuclei), which is a hallmark of the switch from a proliferative state (hyperplasia) to hypertrophic growth (cells increase in size rather than number). The information about ESCs and differentiation stages was adapted from [35,36,37,38].

In vertebrates, the heart is a complex organ composed of multiple diverse cell types, including cardiomyocytes, conduction system cells, endothelial cells, vascular smooth muscle cells, and cardiac fibroblasts [29,30]. The proper organization and localization of those cells within the heart are orchestrated by complex differentiation and patterning processes during embryogenesis. In this section, we will focus on the major steps of heart development taking place from embryonic day (E)16 to E60 in humans and E7.5–E15 in mice [31,32]. All resident cells of the heart are derived from distinct cardiac progenitor cells (CPCs) that are spatially and temporally segregated in the developing embryo [1,33]. The majority of cardiac cells arise from the mesodermal layer, which is initially characterized by the expression of the transcription factor Brachyury/T [34]. Some of these mesoderm cells are further specified into early cardiac mesoderm precursor cells expressing the transcription factor Mesp1 (Figure 1).

Around mouse E6.5–7.0 (human E21–28), Mesp1-positive cells exit the primitive streak and rapidly originate two distinct populations of CPCs called the first heart field (FHF) and second heart field (SHF). The FHF is marked by the expression of the transcription factors Nkx2.5 and Tbx5, whereas the transcription factor Isl1 is specifically expressed in a subset of Mesp1-derived cells contributing to the SHF [39]. By mouse E8.5 and around human E20, cells of the FHF form the cardiac crescent and next migrate and fuse at the midline of the embryo to form the primitive linear heart tube. This structure is composed of an outer (myocardium) and an inner cell layer (endocardium), separated by an extracellular matrix (ECM) called the cardiac jelly. The linear heart tube contributes to the formation of the future left ventricle and atrial chambers [34,40]. By mouse E8.0 and human E21–22, the heart tube initiates rhythmic contractions due to cardiomyocyte activity and undergoes a rightward looping at around mouse E9.0 and human E24–28 [34,40,41,42]. The SHF progenitors, on the other hand, which are initially present medially and posteriorly to the cardiac crescent (Figure 1), migrate into the linear heart tube, extend into the pharyngeal mesoderm, and participate in the looping heart tube. CPCs of the SHF also contribute to the formation and elongation of the outflow tract (OFT), right ventricle, atrium, venous pole of the heart, and the primary atrial septum [34,40,41,42]. At this stage, another type of progenitor, the proepicardium cardiac progenitors, migrates onto the outer cardiac surface to produce the cells of the epicardium which cover the myocardium [43]. The sinus venosus comprising the sinoatrial node and the sinus horns derives from a distinct population of cardiac precursors expressing Tbx18 [34,44]. Some epicardial cells undergo an epithelial-to-mesenchymal (EMT) transition and originate epicardium-derived progenitor cells, which migrate into the heart and differentiate into smooth muscle cells, cardiac fibroblasts, and endothelial cells, which contribute to the coronary vasculature [43].

By E9.5 in mice and E34 in humans, endocardial cushions form in the atrioventricular canal (AVC) and OFT through migration of endocardial cells between the endocardium and myocardium [45]. These structures are predominantly composed of mesenchymal cells generated via endocardial EMT [46]. The muscular interventricular septum develops from the myocardium at the caudal end of the forming ventricle, starting around mouse E10.5 and human E52, and will separate the right and left ventricles [32,34]. Atrial septation begins at approximately mouse E10.5–E11.5 and human E32–34 [32]. During this process, the septum primum and septum secundum (membranous structures growing from the roof of the primitive atrium) extend toward the developing endocardial cushions. These structures partially divide the common atrium and leave an opening, the foramen ovale, which allows oxygenated foetal blood to flow from the right atrium to the left atrium. Normally, the foramen ovale closes soon after birth, and failure of this closure results in an atrial septal defect (ASD). At the same time, the common OFT is divided into the aorta and pulmonary artery, a process requiring contributions from cardiac neural crest cell progenitors, which migrate from the neural tube by mouse E9.5 and also participate in remodelling the pharyngeal arch arteries, valvulogenesis, and the development of the cardiac conduction system [34,47]. Atrioventricular valvulogenesis occurs around E14.5–E17.5 in mice and E44–E60 in humans [31,47].

Thus, the intricate orchestration of cardiac development involves the precise timing and coordination of multiple cell lineages, including mesodermal progenitors, neural crest cells, and epicardium-derived cells, to form the heart’s chambers, valves, and great vessels. Disruptions in the coordinated cellular and molecular events of heart development may lead to CHDs, highlighting the importance of these processes for proper cardiac function.

## 3. Tracing the Role of CITED in Heart Development

Cited1 protein expression was detected in early mouse embryos from the two-cell to the blastocyst stage and primarily localized in the cytoplasm [48]. At later stages, *Cited1* is expressed in cultured trophoblast lineages and in the trophectoderm (TE) of early mouse embryos at higher levels than in the inner cell mass (ICM) cells [48]. *Cited1* depletion in mouse ESCs led to a compromised trophoblast differentiation induced by Bmp4, while its overexpression in those cells induces a trophoblast-like state through activation of the BMP signalling pathway. Additionally, Cited1 overexpression upregulated trophoblast signature genes while downregulating pluripotency-related genes, indicating that Cited1 promotes trophectodermal differentiation rather than ICM identity [48]. *Cited2* is expressed in both trophectoderm- and mesoderm-derived placental cells, with particularly high expression levels in the junctional zone of the placenta and invasive trophoblast cells that migrate from this zone into the uterine parenchyma in rodents [14,16,17]. Similarly, in humans and rats, CITED2 is present in extravillous trophoblasts, where it plays a critical role in placental development and function [16]. In addition, in mice, *Cited2* disruption leads to smaller, poorly vascularized placentas with reduced numbers of differentiated trophoblast cell types, resulting in placental insufficiency and embryonic lethality [14,17].

**Figure 2:**
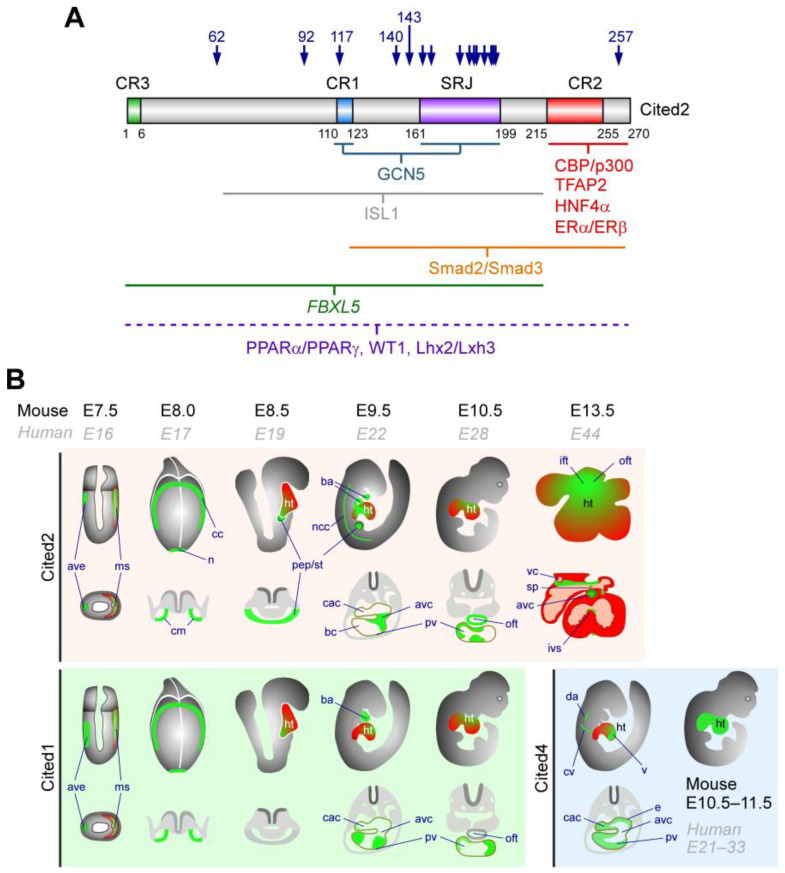
CITED interaction domains and expression during mouse embryonic development. (**A**) Representation of the human CITED2 protein showing the conserved regions (CR13). CR2 is shared by all CITED proteins, while CR1 is common to CITED1 and CITED2, and CR3 is found in CITED2 and CITED4. The serine-glycine-rich junction (SRJ), unique to CITED2, is also indicated. CITED2 protein interactors and their interaction domains with CITED2 are indicated below the CITED2 diagram. The CITED2 domains and its interacting proteins are indicated. Blue arrows mark the locations of CITED2 variants identified in CHD patients. (**B**) Illustrations of the temporal and spatial expression of *Cited2* transcripts represented by green areas (top panel) in cardiac-related regions with the corresponding transverse sections below. At E7.5, Cited2 is expressed in the anterior visceral endoderm (ave) and mesoderm (ms), and at E8.0 in the cardiogenic mesoderm (cm), cardiac crescent (cc), and node (n). From E8.5 to E9.5, its expression is detected in regions containing cardiac progenitors of the proepicardium (pep) and hepatic progenitors of the septum transversum (st). At E9.5, *Cited2* expression extends to the myocardium near the proepicardium, including the outflow tract (oft), bulbus cordis (bc), primitive ventricle (pv), common atrioventricular canal (cac), cardiac neural crest cells (ncc), brachial arches (ba), and atrioventricular canal (avc). By E10.5, *Cited2* is detected in the trabeculae of the primitive outflow tract (oft) and the myocardium of the common ventricular chamber (pv). At E13.5, low transcripts levels are found throughout the myocardium of the prospective left and right atria and ventricles, with stronger expression in the septum primum, endocardial cushion tissue in the AVC, and around the vena cava (vc). Additional labelled structures include the common cardinal vein (cv), dorsal aorta (da), ventricles (v), atria (a), superior part of the atrial septum (sp), intraventricular septum (ivs), and endocardium (e). At later stages, Cited2 expression becomes more homogeneous throughout the heart [11,13]. The bottom left panel indicates *Cited1* expression from E7.5 to E10.5, and the bottom right panel indicates *Cited4* expression from E10.5 to E11.5, adapted from [13,49,50].

Prior to gastrulation at mouse E5.5 (Figure 2), *Cited2* is expressed in the anterior visceral endoderm (AVE), while *Cited1* is detected in the embryonic visceral endoderm and the visceral yolk sac endoderm [49]. The AVE derives from the visceral endoderm and produces secreted factors to signal the epiblast and orchestrate the correct primitive streak formation, anterior patterning of the embryo, and cardiac development. Later during gastrulation, *Cited2* expression is observed in the anterior mesoderm located next to the AVE (Figure 2B). At mouse E7.0–E7.5, in the cells that derive from the epiblast, *Cited1* transcripts are first detected in the nascent mesoderm adjacent to the primitive streak, while *Cited2* is concurrently expressed in the ventral node and cardiogenic mesoderm [13,49]. Next, *Cited1* and *Cited2* expression is maintained throughout the cardiac crescent and in the myocardium (Figure 2B). At E8.5, *Cited2* is broadly expressed throughout the myocardium of the primitive heart tube of mouse embryos and is essential for early cardiac morphogenesis. In contrast, *Cited1* is present in the developing heart at this stage but exhibits a more restricted and lower expression pattern than *Cited2* [13,49]. The precise localization of *Cited1* expression within the heart tube is less characterized than the expression of *Cited2*, and its role in cardiac development appears to be less critical than that of *Cited2* [13,49]. Between E8.5 and E9.5, *Cited2* expression is detected in the presumptive septum transversum and the first and second branchial arches [13,49]. *Cited2* expression is also high in CPCs of embryonic hearts at E9.5, particularly cardiac progenitors of the SHF [22]. The function of *Cited2* in CPCs remains to be elucidated.

At early stages (E8.0), *Cited4* transcripts are only detected in the yolk sac and in the blood islands, but by E9.5 they are present in the cardinal vein and dorsal aorta, and in the developing atria and ventricles, particularly in the endocardium [18], and persist in the whole heart by E10.5 and E11.5 [50]. Between E9.5 and E10.5, Cited2 expression is broadly distributed and highly enriched within the developing cardiac structures (Figure 2B), including the aortic sac, the compact myocardium and trabeculae of the ventricles and the myocardium adjacent to the endocardial cushions of the OFT, the AVC, the presumptive atria, the inflow tract (IFT), and in the fourth branchial arch [13,49]. *Cited1* transcripts are also present in the future presumptive right ventricle and atrium by E10.5 to E12.5 [49]. By E13.5, *Cited2* expression is primarily confined to the OFT, the IFT, the septum primum, regions surrounding the vena cava, the endocardial cushions of the atrioventricular canal, and the apex of the interventricular septum [13].

Despite the considerable overlap in expression among *Cited1, Cited2*, and *Cited4* within cardiogenic regions during mouse embryogenesis, only embryos carrying a germline *Cited2* knockout exhibit severe developmental impairments and die during gestation with a myriad of cardiac abnormalities and defective establishment of the left–right axis, which also contributes to correct heart formation [5,9,10,11,12,13,14,15,17,18,51,52,53,54,55,56,57,58].

On the other hand, *Cited1*-null mice die immediately after birth due to defects in trophoblast-derived extraembryonic tissues, while *Cited4*-null mice are viable, but no apparent cardiac defects were observed in mice lacking *Cited1* or *Cited4* [18,58]. Interestingly, *Cited2* is also required for trophoblast proliferation and differentiation, as well as normal placental development and vascularization in mouse models [14,17,59]. Consequently, Cited2 dysfunction may simultaneously compromise both cardiac and placental development, further exacerbating cardiac defects and embryo outcomes.

In mammals, during foetal development and the first week after birth, cardiomyocytes are generally mononucleated with a high proliferative potential to allow the growth of heart tissue, thereby providing most of the cardiomyocytes necessary throughout the lifetime of the organism [60]. The transition from mononucleated to binucleated (or multinucleated) cardiomyocytes occurs by uncoupling of DNA synthesis/nuclear division from the cytoplasmic scission and marks the cardiomyocytes’ terminal differentiation stage and loss of proliferation. Thus, subsequent increases in heart size, due to physiological or pathological stimuli, will occur by expansion of the size of the cardiomyocytes, a process termed hypertrophy [60].

The function of *Cited2* in the heart after birth and throughout adult life is still poorly understood. However, blocking *Cited2* expression in neonatal rat cardiomyocytes through miR-410 and miR-495 leads to increased DNA synthesis and proliferation via downregulation of the p57 cyclin kinase inhibitor [61]. Since Cited2 is critical for heart development in mice, its role in postnatal rat cardiomyocytes is unexpected. Further studies could clarify whether Cited2 expression functions to preserve cardiomyocytes as mononucleated rather than allowing binucleated conversion. On the other hand, Cited4 overexpression in primary cardiomyocytes drives their proliferation and heart hyperplasia, at least in part through increased expression of cyclin D1 [19]. Thus, Cited2 and Cited4 appear to have distinct and perhaps antagonistic roles in regulating postnatal cardiomyocyte proliferation. Unlike *Cited2* and *Cited4* expression, abundant in mouse and human adult hearts, *Cited1* expression is absent, and its role in adult hearts is negligeable [6,8,18,26,27,28]. Interestingly, the decrease in CITED2 expression has been associated with impaired cardiac function in human ischemic cardiomyopathy, emphasizing its potential importance in disease mechanisms and as a possible therapeutic target [62].

A recent study has shown that Cited2 is essential for maintaining the regenerative capacity of mouse cardiac stem cells during ageing and highlighted its potential as a therapeutic target for enhancing cardiac repair in the ageing heart [63]. However, prevailing evidence supports that heart regeneration in mammals, when it occurs, is primarily mediated by the proliferation and, in some cases, dedifferentiation of existing cardiomyocytes, not by progenitor cells [64,65]. Future research should focus on elucidating the precise molecular mechanisms by which CITED2 and CITED4 regulate cardiomyocyte function, and on exploring their potential as therapeutic targets to enhance cardiac repair and regeneration in heart disease.

## 4. Molecular Mechanisms of CITED-Mediated Cardiac GENE Regulation

### 4.1. CITED Proteins and Interacting Partners

At the molecular level, CITED2 strongly binds to transcriptional co-activators and histone acetyltransferases CBP and p300 (CBP/p300), which have also been linked to CHDs in Rubinstein–Taybi syndrome [8,66,67]. Three conserved regions (CR1–3) were described in CITED proteins (Figure 2A). A specific SMAD4-Interaction Domain (SID) has also been evidenced in CITED1 [68]. The CR2 domain is the hallmark of the CITED proteins, which encompasses the CBP/p300 binding domain and has been clearly identified as a potent transactivation domain. CITED proteins exhibit a high binding affinity to the cysteine-histidine-rich domain 1 (CH1) of CBP/p300. Binding of CITED proteins to CH1 may outcompete other transcription factors such as HIF-1α for the binding to CBP/300 and consequently repress the HIF/hypoxia signalling pathway [6,7,8,68,69,70]. In addition, *CITED2* is itself a hypoxia/HIF-target gene, and its expression may protect cells and embryos from an excessive hypoxic response [8,15]. CITED2 also acts as a negative regulator of transcription factors including RXRα, NF-κB, STAT2, p53, and ETS-1, all of which interact with the CH1 domain of CBP/p300 [8,71,72,73,74,75,76]. Conversely, CITED2 functions as a co-activator for numerous transcription factors that depend on CBP/p300 cooperation for transcriptional efficiency, including TFAP2 members, LHX2/3, SMAD2/3, PPARα/γ, HNF4α, WT1, GCN5, and ISL1 [5,9,22,77,78,79,80,81,82,83,84,85]. CITED2 also promotes PGC-1a activation of the hepatic gluconeogenic programme upon glucagon stimulation via prevention of its GCN5-mediated acetylation during fasting, while insulin disrupts the GCN5–CITED2 interaction, suppressing PGC-1a activity to aborting the gluconeogenic programme [83].

The CITED proteins also contain a conserved CR1 motif, which is present in CITED1, CITED2, and CITED3 and is located centrally within these proteins, as well as a shorter CR3 motif at the N-terminus of CITED2, CITED3, and CITED4 [86]. Moreover, CITED2 in placental mammals possesses a unique, serine-glycine-rich junction (SRJ) domain, which is highly conserved and represents a hotspot for mutations associated with CHDs [25]. Overall, the precise function of these conserved domains and SRJ remains largely unknown.

### 4.2. Regulation of CITED Expression

Little is known about the stimuli and pathways that govern the expression and function of the CITED family members during embryonic development. However, the cellular expression of Cited2 is highly sensitive to numerous extrinsic and intrinsic cellular signals, including conditions like hypoxia through a direct transactivation of HIF-1α and Forkhead box (Fox) factor FOXO3A, ischemia, interleukins, adrenocorticotropic hormones, growth factors such as PDGF, GM-CSF, TGF-β, β-FGF, and NGF, hemodynamic shear forces, oestrogens, interferon-α, insulin, elevated glucose levels, lipopolysaccharides (LPS), mechano-transduction triggered by primary cilia due to strain, indoxyl sulfate, endoplasmic reticulum stress, and DNA damage stimuli [8,73,81,82,87,88,89,90,91,92,93,94,95]. In addition, Foxp1 stimulates *Cited2* expression and the expression of other transcription factors required for pluripotency in mouse ESCs [23,96]. *Cited2* transcription within the mesoderm is upregulated by signalling through Wnt3/WNT3A [97]. In endothelial cells, the active insulin-receptor/PI3K/Akt pathway inhibits FoxO1 activity, leading to decreased expression of CITED2 [92], while *VEGF* modulates CITED2 expression in human coronary artery cells through the control of FoxO1 activity [98]. Additionally, FoxO3 has been shown to directly induce Cited2 expression in normoxic and hypoxic conditions in erythroid progenitors and mouse embryonic fibroblasts [95,99]. Interestingly, *Cited2* may also be upregulated by FoxO1 and FoxO3 in cardiomyocytes during heart infarction [100]. In contrast to Cited2, the regulatory signals and stimuli that control the cellular expression of Cited1 and Cited4 remain largely uncharacterized.

Cited1 co-activates Smad4 upon TGF-β/BMP pathway activation and represses the Wnt/β-catenin signalling pathway [101]. The regulation of *Cited1* expression by hypoxia in mammals has not been established, but its expression is upregulated by Wnt3a in ESCs and by the parathyroid hormone in osteoblast-lineage cells [102,103]. During mouse embryogenesis, Cited1 and Hand1 show overlapping expression domains, particularly within trophoblast and ventricular regions of the developing heart [49,58,104]. Compared to wild-type embryos at E9.5–10.5, *Cited1* expression was strongly reduced in the hearts of *Neuregulin* (*Nrg*)1-null embryos at E8.5 and in the left cardiac ventricle of *Nrg1-*, *Tbx20*-, and *Hand1*-null embryos [104,105,106]. These observations argue that Nrg1 (through the ERK1/2 MAP kinase pathway), Tbx20, and Hand1 act upstream of *Cited1* during cardiac development, but the exact contribution of *Cited1* downregulation to the phenotypes observed in *Nrg1-*, *Tbx20*-, and *Hand1*-null embryos remains to be clarified. *Cited4* expression is induced by training exercise in adult hearts [19,107].

Post-transcriptional and post-translational regulatory mechanisms have also been shown to modulate CITED expression. For example, TGF-β activation accelerates the degradation of *Cited2* mRNA after transcription, reducing its stability [108]. The binding of CITED1 to p300 and the co-activation of Smad4 are regulated by phosphorylation of CITED1 residues during the cell cycle [68]. In vitro studies indicated that CITED2 can be phosphorylated by MAP kinases, although the significance of this modification for its cellular and developmental functions remains unclear [25]. Recently, a non-synonymous CITED2 mutation, p.Pro101Ser, identified in a patient with CHD, was shown to increase CITED2 transcriptional activity at several downstream target genes important for cardiac development, likely due to an altered phosphorylation pattern of the mutant protein [109]. Additionally, at the protein level, CITED2 stability appears to be tightly controlled through proteasome-dependent degradation under conditions such as normoxia, hypoxia, and varying cellular iron levels [69,110]. These findings underscore the importance of post-translational regulatory mechanisms acting on CITED2 for proper heart development. However, the post-translational regulation of Cited proteins remains underexplored.

### 4.3. CITED Proteins and Cardiogenesis

In vitro differentiation of mouse ESCs used as a model to recapitulate cardiogenesis (Figure 1) has revealed that the loss or downregulation of *Cited2* expression in ESCs during the early stages of differentiation drastically impaired the cardiogenic process [22,111]. This observation is consistent with in vivo experiments that have indicated that *Cited2* conditional-knockout in either Brachyury/T- or Mesp1-expressing cells does not overtly affect heart development, suggesting that *Cited2* may play a major role in cardiogenesis before mesoderm specification [10]. Consistently, during mouse embryonic development, *Cited2* is highly expressed in early mesodermal cardiac Mesp1-positive progenitors in the earliest Tbx5-positive CPC of the FHF isolated from embryos prior to Nkx2.5 expression [112,113]. In addition, mesoderm progenitors that originated from human ESCs or induced pluripotent stem cells (iPSCs) reprogrammed from somatic cells also express CITED2 [114], suggesting a conservation of CITED2 function in mouse and human cells. In contrast to *Cited2*, which is present in ESCs and maintained during the entire differentiation process [22], *Cited4* expression emerges in ESC-derived cells after the transient expression of *Brachyury/T* [115]. Whether Cited4 can compensate for the absence of Cited2 activity after mesoderm specification, either in vitro or in vivo, remains to be determined.

The mechanisms by which *Cited2* exerts its functions at this stage remain unknown. However, depletion of *Cited2* at the onset of ESC differentiation leads to a marked downregulation of *Brachyury/T*, *Mesp1*, *Isl1*, and to a lesser extent *Gata4*, *Nkx2.5*, and *Tbx5* expression, as well as reduced levels of mediators of cardiogenic signalling pathways, such as Wnt5a and Wnt11 proteins (Table 1). Additionally, Cited2 is expressed in committed ventricular progenitors of the SHF co-expressing Isl1 and Nkx2.5 in developing hearts at E9.5 [22], but no major heart defects were observed in embryos with *Cited2* depletion from progenitors of the FHF and SHF and cardiac neural crest cells [11,12]. The role of Cited2 in embryonic cardiac progenitors remains elusive.

During mouse embryonic development, defective regulation of *Nodal*, *Lefty1/2*, *Pitx2c*, and *Vegfa* expression due, in part, to impaired cooperation among Cited2, Tfap2a, and CBP/p300 (Table 1) has been proposed to contribute to both the cardiac malformations and disrupted establishment of left–right asymmetry observed in *Cited2*-null mouse embryos [5,9,10,13]. The Tfap2a–Cited2 complex activates the P1 promoter of Pitx2c and promotes cardiac *Vegfa* expression during mouse embryo development [9,10], while Cited2 may cooperate with Smad2/3 and Foxh1 to enhance *Nodal* transcription via its Asymmetric Sequence Element [12]. In addition, Cited2 directly regulates *Isl1* expression, physically interacts with ISL1, and the CITED2–ISL1 synergizes to promote mouse ESC differentiation toward cardiomyocytes [22]. Interestingly, the proper regulation of *Isl1* is critical for SHF-derived cardiac structures, and its dysregulation could account for the cardiac malformations observed in *Cited2*-null embryos [5]. Vascular endothelial zinc finger 1 (Vezf1), which is important for embryonic vasculature formation, may exert its role by modulating the expression of *Cited2* [116]. Collectively, these observations indicate that *Cited2* holds a critical and unique function in cardiovascular development that cannot be compensated for by other members of the *Cited* gene family.

## 5. *CITED2* Mutations and Congenital Heart Disease

**Figure 3:**
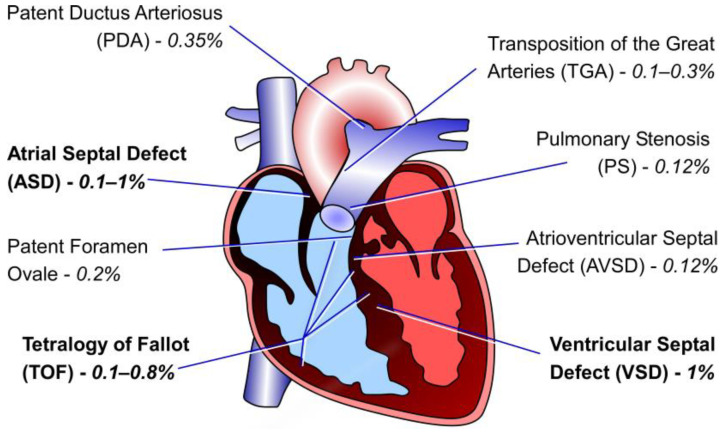
Illustration of the adult human heart highlighting the cardiac defects observed in CHD patients with CITED2 mutations. The percentages indicated correspond to the frequency of each defect associated with CITED2 mutations within the studied patient groups. Data was acquired from the published studies [25,118,119,125].

Worldwide genetic screens of patients with CHDs have identified synonymous and non-synonymous variants in *CITED2* associated with sporadic non-syndromic CHDs [25,117,118,119,120,121,122,123,124]. Thus, CITED2 is considered a useful marker for prenatal diagnosis of CHDs [121]. The cardiac defects observed in mouse *Cited2*-null embryos are phenotypically comparable to the heart abnormalities reported in patients carrying *CITED2* mutations (Table 2). Ventricular (VSD) and atrial (ASD) septal defects, transposition of the great arteries (TGA) and tetralogy of Fallot (TOF) are the most frequent heart anomalies associated with *CITED2* dysfunction (Figure 3). Notably, most of missense mutations are concentrated within the SRJ domain, a region exclusive to CITED2 (Figure 2A). The SRJ domain is predicted to be highly unstructured, and its function remains elusive since transgenic mice expressing solely the human CITED2 and lacking the SRJ domain are viable with normal heart features [25].

The majority of CITED2 mutations identified in CHD patients have minimal impact on CITED2′s ability to inhibit HIF-1α-driven transcriptional activity or to enhance TFAP2 transcriptional activity ex vivo [25,117,118,119,120,121,125]. Furthermore, mutations in *CITED2* identified in patients can cause significant disruption of *VEGF* expression, essential for proper vascular formation, as well as reducing the expression of the transcription factor *PITX2C*, which is critical for left–right body patterning and cardiac development [118]. Alternatively, mutations of *CITED2* may result in hyperactivity of HIF-1α and also hinder cardiogenesis in mice [15]. Considering that CITED2 interacts with key transcription factors critical for heart development, including ISL1 and SMAD2/3, it is important to assess their transcriptional defects alongside mutations in *CITED2.* Altogether, the evidence above suggests that the multifunctional roles of CITED2, when altered by mutation, may impact normal cardiac development in a non-linear manner.

The haploinsufficiency of the *Cited2* gene has been revealed in mice, since a significant part of the heterozygous embryos (harbouring both a *Cited2* functional allele and a Cited2-*null* allele, expressing half the normal levels of *Cited2* transcripts) displayed cardiac malformations [11]. This indicates that, beyond alterations in the CITED2 protein sequence or function, a reduction in CITED2 protein levels caused by genetic or epigenetic deregulations, increased protein instability, or degradation during gestation may increase the penetrance of CHDs and perhaps make embryos more sensitive to environmental stress. Thus, the three *CITED2* variants identified within the 5′-untranslated region (UTR) of the exon 1 and 3′-UTR of the exon 2, associated with ASD, VSD, and TOF [125], and another study associating decreased *CITED2* expression to abnormal hypermethylation of CpG islands on its promoter in children with CHD [117] may be a consequence of *CITED2* haploinsufficiency. Abnormal hypomethylation in the upstream regulatory regions of *CITED1* has been observed in monozygotic twins affected by double-outlet right ventricle. However, the contribution of *CITED1* to the disease aetiology remains to be clarified [126]. Overall, these observations argue in favour of the assessment of CITED2 expression levels and their impact in CHDs as a meaningful approach in both research and clinical contexts.

The embryonic and foetal environment also has a critical impact on heart development, as inadequate placental function, sporadic developmental errors, and/or maternal environmental factors, such as diabetes, some medications and drugs abuse, infections, tobacco smoke, alcohol, and harmful chemicals, are known to increase the risk of CHDs [1,127]. Interestingly, the combination of maternal high-fat diet together with embryonic *Cited2* deficiency resulted in a drastic increase in the occurrence of defects in left–right body axis patterning and heart abnormalities in comparison to *Cited2*-null embryos of gestating mice fed with a normal diet [128]. Moreover, maternal diabetes in rats results in VSD and cardiac thin ventricular walls, with embryonic hearts displaying a concomitant decrease in both *Cited2* transcript and protein expression levels [129]. Considering these observations, maternal high-fat diet and/or maternal metabolic adverse conditions such as diabetes are likely to interplay with foetal *CITED2* expression in human gestation and predispose for CHDs, particularly if the foetuses harbour mutations in the *CITED2* gene. The downregulation of *CITED2* expression by maternal diabetes/high glucose may be controlled through miR-200b, which interferes with the cardiogenic processes and targets *CITED2* [130]. On the other hand, gene expression analysis of mouse embryos exposed to maternal diabetes showed a two-fold increase in *Cited4* transcript levels in comparison to wild-type embryos [131]. The significance and the importance of diabetes-induced *Cited4* transcripts in heart development remain to be established. However, diabetes-induced *Cited4* expression is unlikely to compensate for the downregulation of *Cited2*, further indicating that *Cited* genes have distinct functions.

In vitro exposure of human cardiomyocytes to high glucose levels downregulates *CITED2* expression and increases apoptosis, while the overexpression of wild-type CITED2 confers an efficient protection to cardiomyocytes against apoptosis. Conversely, the overexpression of CITED2 harbouring the p.ser192gly mutation failed to be as effective [129], indicating that mutations of *CITED2* may affect important cellular processes, such as the support of cardiomyocyte survival under stress conditions.

Pre-eclampsia is a severe pregnancy complication characterized by placental dysfunction and reduced blood flow, which limits oxygen and nutrient delivery to the foetus and compromises foetal development of the cardiovascular system and increases the risk of CHDs. Interestingly, CITED2 expression is elevated in placental tissues from pre-eclamptic pregnancies compared to controls. Since CITED2 promotes trophoblast proliferation, migration, and invasion, while suppressing apoptosis during placental development, its increased expression in pre-eclampsia may represent a protective or compensatory mechanism to maintain placental integrity by enhancing trophoblast cell survival and invasiveness [132,133]. It would be of interest to investigate whether *CITED2* mutations are associated with a poor outcome in pre-eclamptic gestations.

Sequence variants in the *CITED2* promoter of patients with VSD or ASD displayed a significant reduction in their activity in transient transfection assays compared to control promoters [134,135]. Notably, one of these variants which disrupts an SP1 binding site is critical for CITED2 expression [134], indicating that promoter variants may create or disrupt binding sites for transcription factors, which will influence the levels of *CITED2* transcripts expressed in the cells. Surprisingly, a non-synonymous mutation, p.Pro101Ser, identified in a patient with VSD, increased CITED2 activity resulting in the upregulation of several downstream cardiac developmental genes, including G*ata4*, *Mef2c*, *Nfatc1 and Nfatc2*, *Nodal*, *Pitx2*, *and Tbx5* [109]. Furthermore, two synonymous variants were found to affect the mRNA structure and translation efficiency of *CITED2* transcripts [109].

Overall, these observations indicate that both loss and gain of *CITED2* function can disrupt cardiac development, highlighting the critical importance for precise regulation of its expression and activity. Thus, proper timing and controlled levels of *CITED2* expression are crucial to ensure the balanced activation of downstream cardiac genes during development. Any deviation, whether reduced or excessive CITED2 activity, may destabilize this regulatory network and contribute to CHDs. In addition, *CITED2* may be essential to resist environmental stresses and maintain placental function. By supporting these processes, CITED2 may also support normal embryonic development, and disturbances in its function could increase susceptibility to CHDs.

## 6. *CITED* Genes in ADULT Heart Physiological Adaptations and Protection Against Pathological Stress

*CITED2* and *CITED4* are both expressed in adult hearts, but their specific contributions to maintaining heart homeostasis or their roles in adult cardiac pathological processes are not yet fully understood. However, patients with ischemic cardiomyopathy or a cardiomyopathy caused by infection with the protozoan *Trypanosoma cruzi* (Chagas disease) exhibit lower CITED2 transcript levels compared to control individuals. Among these patients, higher CITED2 expression is also associated with an increased left ventricular ejection fraction, a key indicator of cardiac performance and overall heart health [62,136]. In H9C2 rat cardiomyocytes subjected to hypoxia-reoxygenation, an in vitro model of ischemia–reperfusion (I/R) injury, *CITED2* expression was significantly reduced [137]. Thus, I/R injury may actively downregulate *CITED2* expression in ischemic hearts, which likely exacerbates the chronic maladaptive activation of the HIF pathway, a key mediator of I/R injury and the HIF-driven metabolic switch. Such dysregulation is likely to contribute to cardiac degeneration and heart failure progression by promoting energy deprivation [62,138]. Conversely, the overexpression of *CITED2* reduces cardiomyocyte pyroptosis (a form of inflammatory programmed cell death) by decreasing the activation of pyroptotic markers and lowering the secretion of inflammatory cytokines such as IL-1β and IL-18, primarily through the suppression of HIF-1α activity [137]. The protective role of *CITED2* in myocardial I/R injury is also closely linked to its regulation by FoxO transcription factors, particularly FoxO1 and FoxO3, which are critical mediators of oxidative stress resistance in cardiomyocytes, promoting cell survival by inducing antioxidant enzymes, anti-apoptotic proteins, and autophagy-related genes [139]. Importantly, FoxO factors directly activate CITED2 expression, which, in turn, inhibits HIF-1α transactivation, thereby reducing pyroptosis and inflammation in ischemic cardiomyocytes [100]. Thus, CITED2 is a key downstream effector of FoxO-mediated cardioprotection by modulating hypoxic responses, limiting inflammatory cell death, and enhancing survival pathways under conditions of oxidative stress. This coordinated FoxO/CITED2/HIF-1α axis highlights an important molecular mechanism by which the heart adapts to I/R stress, offering potential therapeutic targets for reducing myocardial injury. Together, these observations argue that *CITED2* expression preserves the function of adult hearts during adverse ischemic cardiomyopathy. However, in cardiac arterial endothelial cells of patients with type 2 diabetes, and in insulin-resistant or obese animal models, *CITED2* expression is increased compared to control individuals [92]. In this context, elevated CITED2 expression may inhibit HIF transactivation, thereby limiting HIF’s pro-angiogenic function and impairing the adaptation of endothelial cells to hypoxia and hyperglycaemia, conditions commonly associated with diabetic complications [92]. Thus, the role of CITED2 in cardiomyocytes, where it supports cell survival and regulates metabolism, and in endothelial cells, where it inhibits angiogenesis and inflammation, illustrates the complexity of its function in heart tissue. This complexity also emphasizes the importance of considering specific cell types when evaluating CITED2 as a potential therapeutic target in heart disease.

Both iron deficiency and iron overload may impact cardiac function and are considered age-aggravated risk factors for cardiovascular disease [140]. Interestingly, FBXL5, which is an E3 ubiquitin ligase considered as an oxygen and iron sensor, targets CITED2 protein for proteasome-dependent degradation in normoxic conditions [69]. Since FBLX5 affects CITED2 expression levels, it would be of interest to investigate the impact of FBXL5 activity in heart function/protection during ageing or upon insults resulting from systemic or cellular iron content dysregulations.

On the other hand, CITED4 is a key regulator of exercise-induced physiological cardiac hypertrophy and cardiomyocyte proliferation in mice [19,107]. Indeed, exercise upregulates CITED4 expression, which promotes cardiomyocyte survival, physiological hypertrophy, and proliferative capacity. CITED4 upregulation is concomitant with the increase in GATA4 expression, a key transcription factor that promotes cardiac regeneration by stimulating cardiomyocyte proliferation, angiogenesis, and pro-regenerative gene expression. This suggests that CITED4 is part of a coordinated regulatory network supporting exercise-induced cardiac growth and regeneration [141]. Interestingly, the ectopic overexpression of CITED4 in cardiomyocytes in adult mice provokes the increase in cardiomyocyte size and heart weight, while preserving systolic function, mimicking exercise-induced effects [28]. Of interest, CITED4 also prevents excessive cardiomyocyte elongation, restraining their size to physiological limits, thereby preventing pathological hypertrophy [19,142]. Moreover, upon ischemic injury, CITED4 activates the expression of genes that facilitate myocardial repair and functional recovery. Although the overexpression of CITED4 does not reduce initial infarct size in I/R models, it attenuates adverse ventricular remodelling and fibrosis, improving long-term survival of animals [28,107]. Thus, CITED4 may be essential for protecting the heart from maladaptive remodelling due to stressors such as pressure overload. CITED4 cardioprotective effects in I/R are also attributed to a reduction in autophagosome accumulation and cardiomyocyte apoptosis after reperfusion [28]. This involves modulation of mTOR signalling, along with the upregulation of anti-fibrotic miR-30d and miR-133b, and the downregulation of pro-fibrotic miR-376c by CITED4, which collectively coordinate cardiomyocyte–fibroblast interactions to limit fibrosis and promote adaptive cardiac growth [143]. Moreover, CITED4 is positively modulated by miR-222 and negatively controlled by the transcription factor C/EBPβ, suggesting that CITED4 expression is tightly regulated during healthy heart hypertrophic remodelling [19,142]. Altogether, CITED4 functions as a critical mediator linking exercise-induced cardiomyocyte proliferation with cardioprotection, underscoring its therapeutic potential in ischemic heart disease.

## 7. Clinical and Translational Applications

Conventional pharmacological and surgical treatments usually aim at reducing and managing cardiovascular dysfunctions, failing to address underlying causes of cardiovascular defects and restore full cardiovascular function. However, significant progress has recently been made in the field of gene therapy and regeneration, with promising future strategies for treating both CHDs and ACDs, and to complement classical approaches.

The complexity of the gene network involved in cardiac development and timing constraints of their expression, and the environmental factors acting on developmental pathways, mean that gene therapy for CHDs is still at an investigational state and largely at preclinical or early research stages. At present, only a few key genes are targeted or compensated by gene therapy approaches for cardiovascular diseases, particularly monogenic and inherited cardiac disorders. Moreover, there are no ongoing clinical trials specifically testing gene therapy for CHDs in humans; all assays are still optimized at the preclinical stage with animal models. Genome-editing techniques based on CRISPR-Cas9 are also being developed to target specific gene mutations, along with the optimization of viral and non-viral gene delivery methods aiming to increase the expression of beneficial genes in affected heart tissues [144,145,146]. However, clinically successful and available therapies based on these approaches are few, although early preclinical successes and proof-of-concept clinical trials demonstrate feasibility, particularly for monogenic forms of CHD.

In ACDs, gene therapy approaches are also developed and focus on delivering genes for pro-regenerative or angiogenic factors, such as VEGF or FGF using viral vectors, nanoparticles, plasmids, or modified RNA, showing safety and some functional benefits in trials for conditions such as refractory angina [146,147]. Remarkably, an efficient gene delivery of CITED4 by AAV9-based viral vector in wild-type mice was successful and showed an increase in CITED4 levels in the heart, resulting in beneficial physiological heart hypertrophy without adverse effects [148]. This approach resulted in a decrease in cell death, fibrosis, and inflammation in mice upon I/R injury, leading to smaller scars and improved heart function in treated mice with ectopic CITED4 [148]. These encouraging results demonstrate that future gene therapy based on CITED4 expression could be translated into clinical set-ups to enhance cardiac repair and resilience following ischemic injury in humans. Since CITED2 also presents beneficial properties for heart function and protections, which may be complementary to those of CITED4, it would be interesting to develop similar approaches with CITED2 alone or in combination with CITED4. Nevertheless, durable gene expression, precise targeting, and limited immune responses of delivered genes are the main challenge that will have to be addressed in these strategies.

Stem-cell-based therapies also offer promising approaches for treating ACDs like myocardial infarction and heart failure, using sources such as mesenchymal stem cells and iPSC-derived cells, aiming to repair damaged heart tissue, improve cardiac function, and promote new blood vessel formation [149,150]. Although these therapies are not yet standard clinical practice, they hold promise as less invasive, definitive treatment options as ongoing research continues to optimize their safety and efficacy.

The discovery of multiple cardiac progenitor populations emerging during heart development and the existence of a robust network of evolutionarily conserved genes with complementary functions ensures the resilience of heart development and limits the incidence CHDs [1]. For example, mouse embryos can survive and develop normal hearts despite ablation of more than 50% of cardiac progenitors at early developmental stages, demonstrating a remarkable capacity for cellular compensation in the developing embryonic heart [151]. Moreover, emerging evidence indicates that cardiogenesis is driven primarily by intrinsic genetic programmes, which are modulated by extrinsic factors such as extracellular signals that regulate cardiac cell fate, including their spatial positioning and functional specialization, as early as during gastrulation [1]. This concept is further validated by the capacity of pluripotent stem cells to autonomously generate structured cardiac organoids, which are complex three-dimensional cardiac structures generated in vitro, when cultured in the presence of appropriate ECM components and exogenous signalling molecules [152]. Therefore, enhanced understanding of cardiovascular networks and pathways, along with their spatiotemporal dynamics, may facilitate the identification of complementary signalling pathways that could be exogenously activated, through the supplementation of proteins or molecules, to engage alternative cardiogenic mechanisms. Such strategies could serve as safeguard mechanisms to promote proper heart development and reduce the incidence of CHDs [1]. For instance, folic acid and zinc supplements have been shown to reduce the risk of CHDs, and blocking the TGF-β1 signalling pathway reduces HLHS in humans [1]. Heart malformations and mortality observed in mouse embryos lacking *Id* genes were reduced by upregulation of Igf-1 and Wnt5a upon pre-gestational ESC injection to females [1]. Loss of *ISL1* expression in non-human primate embryos and cells leads to disrupted mesoderm formation and lethality, which was partially reversed by the addition of BMP4 [153]. Remarkably, the transient supplementation before mesoderm specification of either 8R-CITED2 (human CITED2 fused to eight arginine residues allowing its diffusion through the cell membranes to the nucleus) or recombinant Wnt5a/Wnt11 proteins corrected the cardiogenic abnormalities caused by *Cited2* loss in mouse ESCs and zebrafish embryos [22,24]. These observations align with findings indicating that *Cited2* function is primarily required from the blastocyst stage to early mesoderm specification, as depletion of *Cited2* in the epiblast (blastocyst stage) of mouse embryos results in in utero lethality and cardiac malformations, and knockout of *Cited2* in mesoderm cells during gastrulation does not affect mouse embryonic development [11,22,24]. Importantly, supplementing Wnt5a/Wnt11 in wild-type zebrafish embryos decreased both natural mortality rates and heart abnormalities relative to untreated controls [24]. This observation suggests that the protective effects of Wnt5a/Wnt11 supplementation may extend beyond *Cited2*-deficient animals, suggesting also the potential for this approach to be used as a prophylactic intervention to improve cardiovascular outcomes more broadly. Given that the cardiogenic role of CITED2 is conserved across vertebrates, from fish to humans, it would be of interest to undertake further research to investigate whether a strategy of supplementation with appropriate exogenous molecules early during gestation could be adapted for mammals, particularly humans, to reduce the incidence and/or severity of CHDs.

## 8. Conclusions and Perspectives

CITED proteins are non-DNA-binding transcriptional modulators exhibiting distinct functional characteristics, including the modulation of specific transcription factors and regulation of distinct sets of target genes. The unifying characteristic of CITED proteins is the ability to bind with high affinity to the CH1 domain of the transcriptional co-activators and histone acetylases CBP/p300 and act either as transcriptional inhibitors or co-activators. In mice, *Cited1*, *Cited2*, and *Cited4* play distinct, stage- and tissue-specific roles in cardiac, placental, and other developmental processes. *Cited2* is essential in the early stages of embryogenesis, such as mesoderm specification, early heart morphogenesis, and placental function, with its loss causing severe cardiac malformations, placental insufficiency, and embryonic lethality. *Cited1* is crucial for trophoblast differentiation but is not essential for heart development. On the other hand, *Cited4* is dispensable during embryogenesis but promotes postnatal cardiomyocyte proliferation and heart growth.

Mutations in the *CITED2* gene, as well as its overexpression or downregulation, have been associated with CHDs in numerous reports, arguing that its function and expression must be tightly regulated for correct heart development. The SRJ domain of CITED2 has been proposed to accommodate mutations affecting heart development in a way that it is still compatible with embryonic life, while mutations occurring elsewhere in CITED2 protein might affect cardiac development and be lethal, just like *Cited2*-knockout is detrimental for mouse development. Thus, *CITED2* mutations in the SRJ domain may not always be causative of CHDs, but they might exacerbate the effect of other combined genetic mutations or environmental stressors to cause CHDs.

At present, all studies have associated CITED2 variants with CHDs in live children and may have overlooked mutations with dramatic consequences for foetal survival and heart development, before CHD detection or testing is feasible, consistent with findings from *Cited2*-null mouse models, which exhibit embryonic lethality. Moreover, CHDs may arise from, or be exacerbated by, underexplored interactions between CITED2 mutations and other genetic or environmental factors. Indeed, environmental factors to which parents are exposed before and during pregnancy, such as a high-fat diet, diabetes, obesity, alcohol consumption, viral infections, air pollution, and deficiencies in riboflavin or folic acid, are likely to influence the expression of genes critical for heart development, thereby increasing the risk of CHDs. Adverse gestational conditions are particularly relevant, as CITED2 misexpression was associated with pre-eclampsia, a severe obstetric complication that may contribute to CHDs. Thus, CITED2 dysregulation may lead to adverse pregnancy outcomes, including foetal death and CHDs due to lack of adaptive changes to unfavourable maternal environmental conditions, such as hypoxia, oxidative stress, pre-eclampsia, infections, and inflammation. In addition, foetal microenvironment, primarily governed by the placenta, plays a crucial role in CHD pathogenesis. Moreover, *CITED2* dysregulation, which disrupts trophoblast function and impairs the placenta’s response to stresses, may contribute to embryonic growth and developmental defects. Many genes implicated in human CHDs are also expressed and have overlapping function in the placenta during development, suggesting that some genes, such as *CITED2*, may play critical roles simultaneously in cardiac and placental development and function, highlighting the interconnected nature of these two processes. Thus, the role of placental insufficiency in exacerbating CHDs should be further explored, particularly in patients which harbour mutations in genes, such as *CITED2*, which have an impact in both heart and placental development and are likely to influence foetal development and contribute to the severity of CHDs by creating an adverse prenatal environment.

Interestingly, the early function of *Cited2* in cardiovascular development may enable the development of prophylactic strategies to overcome its dysfunction through functional complementation and limit occurrences and/or severity of CHDs. Those strategies could involve supplementation with exogenous proteins, such as the engineered form of human CITED2, which can cross cell membranes and localize to the nucleus, or recombinant WNT5A/WNT11 proteins, which are target genes of CITED2. These proteins have been shown to mitigate the cardiogenic defects in *Cited2*-deficient mouse ESCs and zebrafish embryos when supplemented at early stages of differentiation or development. Although the cardiogenic function of CITED2 is evolutionarily conserved, further studies are required to determine whether similar outcomes would be observed in mouse embryos and human cellular models following treatment with these proteins or other agonistic molecules.

Most of the current understanding of CITED proteins in early trophoblast differentiation and cardiac development originates from studies in rodent models. Although CITED2 mutations are consistently associated with CHDs, the specific molecular mechanisms by which CITED2 contributes to the pathological aetiology of CHDs remain largely unresolved. Likewise, the roles of CITED1 and CITED2 in trophectoderm specification and trophoblast development, which are critical processes for placental formation, have been well documented in mice but remain poorly characterized in humans. Several stem-cell-based embryo models have surfaced recently and revolutionized the study of human development by enabling access to previously inaccessible early human developmental stages, particularly blastocyst implantation and trophoblast invasion [29,151,152,153]. Human blastoids derived from naïve pluripotent stem cells faithfully recapitulate blastocyst formation, embryonic lineage specification, and polar trophectoderm patterning, thereby mimicking key early implantation dynamics [154,155]. These blastoid models provide a mean to facilitate mechanistic studies of implantation, lineage segregation, and transcriptional regulation in human systems that are otherwise inaccessible in vivo. They are particularly adapted and valuable for elucidating the roles of CITED1 and CITED2, which are critical for early embryonic and trophoblast development, as well as for CHD pathogenesis. By combining CITED1 or CITED2 gene editing and multiomics approaches in stem-cell-derived 3D cell structures such as blastoids, trophodermic structures, or cardiac organoids, researchers can interrogate gene functions with high precision. Furthermore, blastoids which model trophoblast and epiblast lineage specification as it occurs in the mammalian preimplantation embryo would bridge important gaps left by rodent models. Indeed, these complex stem-cell-derived models recapitulate key features of early developmental stages, including spatial and temporal lineage segregation, thus providing an invaluable platform for mechanistic study. Importantly, blastoid formation presents a precise timeframe during which rescue assays can be performed to recover deficiencies, such as those triggered by *CITED2* deficiency, by supplementation of exogenous molecules, thereby enabling experimental interrogation of gene function within a defined developmental window in a human context.

In the adult heart, *Cited2* supports cardiac health and mitigates damage caused by I/R injury and other conditions, at least in part through anti-inflammatory mechanisms. On the other hand, elevated *Cited2* levels in diabetic endothelial cells may limit angiogenesis, contributing to impaired vascular repair and function in diabetes. Therefore, the beneficial effects of CITED2 depend on tightly regulated expression levels and are context- and cell-type dependent, varying with the nature and severity of cardiovascular injury and the specific heart tissue involved. In normal adult hearts, *Cited4* is a key driver of exercise-induced physiological hypertrophy and cardiac regeneration, through promotion of cardiomyocyte proliferation and survival. Under stress conditions, such as pressure overload or ischemic injury, Cited4 prevents pathological remodelling, supporting myocardial repair and long-term cardiac function and limiting fibrosis. Overall, these findings indicate that *Cited2* and *Cited4* fulfil distinct but essential roles in maintaining adult heart function, enabling adaptation to exercise-induced stress, and defending against cardiovascular damage. However, the intricate and nuanced regulation of these genes, particularly CITED2 with its diverse protein partners, makes it challenging to predict their precise impact on heart disease. Advancing our understanding of the molecular and cell-specific activities of CITED proteins and their interacting partners will be critical for elucidating their contributions to disease mechanisms and protective responses. This knowledge will facilitate novel therapeutic strategies aimed at preventing cardiac dysfunction or restoring normal heart function. The development of mature human cardiac organoids represents a significant advancement in modelling the adult human heart [156]. Recent studies demonstrates that these organoids recapitulate key features of adult cardiac tissue, including multi-lineage cellular composition, improved sarcomere organization, mitochondrial function, and electrophysiological properties, thus enhancing their physiological relevance compared to earlier, more foetal-like models. These organoids do not fully replicate all aspects of adult heart complexity, but they provide a robust platform for investigating adult-like cardiac disease mechanisms, drug responses, and regenerative processes. These mature human organoids could be instrumental to study gene functions such as CITED2 and CITED4 in healthy heart maintenance and in pathological contexts like inflammation and diabetic heart disease, offering potential for translational cardiovascular research and therapeutic discovery.

Overall, the exact functions of CITED proteins across embryonic and mature organisms, particularly within distinct tissues, cell types, and stem cell populations, are not yet fully defined. Advancing research to unravel the molecular and cellular mechanisms of CITED gene activity is essential for clarifying their contributions to cardiac development, disease processes, and protective responses in the heart. This knowledge will also be instrumental in developing novel therapeutic strategies aimed at limiting cardiac dysfunction and restoring normal cardiac function.

## Figures and Tables

**Table 1:** Network of CITED protein interactions within the cardiovascular system.

CITED Protein	CITED-Interacting Protein	Cardiovascular Target Genes	References
CITED1	CBP/p300	Unknown	[7]
CITED2	CBP/p300	*Pitx2c*	[5,8]
	TFAP2	*Pitx2c*, *Vegfa*, Lefty1 *, Lefty2 *, Nodal *	[5,9,10,13,77]
	ISL1	Mefc2*	[22]
	Unknown	*Isl1*	[22]
	Unknown	Brachyury/T *, Mesp1 *, Gata4 *, Nkx2.5 *, Tbx5 *, Wnt5a *, Wnt11 *	[22,24]
	Unknown	*p57*	[61]
	Smad2	Nodal *	[12]
CITED4	CBP/p300	Unknown	[6,70]
	Unknown	Cyclin D1 *	[19]

* A direct interaction and regulation by CITED proteins remains to be established.

**Table 2:** Most prevalent CHD phenotypes in CITED2 mutation carrier patients.

Cardiovascular Anomaly	Estimated Average % with CITED2 Mutations *	% of CHD Patients with CITED2 Variants (Mutated CITED2/Total CHD Patients)	References
All anomalies	~1.53%	~3.33% (4/120)	[125]
		~1.69% (19/1126)	[25]
		~0.71% (5/700)	[118]
		~0.33% (2/605)	[119]
Ventricular Septal Defect (VSD)	~0.94%	~1.02% (4/392)	[125]
		~0.86% (6/700)	[118]
Atrial Septal Defect (ASD)	~0.35%	~1.02% (4/392)	[125]
		~0.16% (1/605)	[119]
		~0.14% (1/700)	[118]
		~0.09% (1/1126)	[25]
Patent Ductus Arteriosus (PDA)	~0.35%	~0.33% (2/605	[119]
Tetralogy of Fallot (TOF)	~0.29%	~0.77% (3/392)	[125]
		~0.16% (1/605)	[119]
		~0.14% (1/700)	[118]
		~0.09% (1/1126)	[25]
Transposition of the Great Arteries (TGA)	~0.17%	~0.26% (1/392)	[125]
		~0.16% (1/605)	[119]
		~0.09% (1/1126)	[25]
Patent *Foramen Ovale* (PFO)	~0.16%	~0.16% (1/605)	[119]
Atrioventricular Septal Defect (AVSD)	~0.115%	~0.14% (1/700)	[118]
		~0.09% (1/1126)	[25]
Pulmonary Stenosis (PS)	~0.115%	~0.14% (1/700)	[118]
		~0.09% (1/1126)	[25]

* Percentages are the average % of CHD patients with CITED2 variants in the different cohorts referenced in the table.

## Data Availability

Not applicable.
